# *Prmt7* Downregulation in Mouse Spermatogonia Functions through *miR-877-3p*/*Col6a3*

**DOI:** 10.3390/life12081194

**Published:** 2022-08-05

**Authors:** Hongmei Gao, Mingrui Zhang, Jiankang Guo, Zhiguo Liu, Fei Guo, Bingyuan Wang, Yulian Mu

**Affiliations:** 1 Institute of Animal Sciences, Chinese Academy of Agricultural Sciences, Beijing 100193, China; 2 College of Animal Science and Technology, China Agricultural University, Beijing 100193, China

**Keywords:** *Prmt7*, miRNAs, germ cell, spermatogonia

## Abstract

Protein arginine methyltransferases 7 (*Prmt7*) is expressed in male germ cells, including primordial germ cells, gonocytes, and spermatogonia. Our previous study demonstrated that *Prmt7* downregulation reduced the proliferation of GC-1 cells (a cell line of mouse immortalized spermatogonia). However, how *Prmt7* regulates spermatogonial proliferation through miRNA and the target gene remains elusive. Here, we experimentally reduced the *Prmt7* expression in the GC-1 cells and subjected them to miRNA sequencing to explore the miRNA profile and its *Prmt7*-responsive members. In total, 48 differentially expressed miRNAs (DEmiRNAs), including 36 upregulated and 12 downregulated miRNAs, were identified. After verifying the validity of sequencing results through qRT-PCR assays in randomly selected DEmiRNAs, we predicted the target genes of these DEmiRNAs. Next, we combined DEmiRNA target genes and previously identified differentially expressed genes between *Prmt7* knockdown and control groups of GC-1 cells, which resulted in seven miRNA/target gene pairs. Among these miRNA/target gene pairs, we further detected the expression of *Col6a3* (collagen type VI alpha 3) as the target gene of *mmu-miR-877-3p*. The results suggested that *Prmt7* downregulation in mouse spermatogonia might function through *miR-877-3p*/*Col6a3*. Overall, these findings provide new insights into the role of *Prmt7* in male germ cell development through miRNA and target genes.

## 1. Introduction

Spermatogenesis, as a highly specialized process in male reproduction, is based on the self-renewal and differentiation of spermatogonia. This process encompasses mitosis from spermatogonia to spermatocytes, followed by meiosis from spermatocytes to spermatids, resulting in haploid spermatids, which undergo a final differentiation into spermatozoa [1]. To date, numerous studies have identified several key regulators of spermatogonial proliferation, such as extrinsic growth factors (including GDNF, EGF, IGF1, and FGF2) and intrinsic genes (including *Plzf*, *Gfra1*, and *Etv5*) [2,3]. However, the underlying molecular regulation of spermatogonial proliferation remains still unclear.

In our previous study, we found that protein arginine methyltransferase 7 (*Prmt7*), the only type III PRMT (a type of PRMT that only catalyzes the formation of monomethylarginine of its protein substrates), can replace *Sox2* in combination with *Oct4*, *Nanog*, and *Klf4* to successfully produce induced pluripotent stem cells [4]. Our further study revealed that *Prmt7* was highly expressed in mouse testes at both mRNA and protein levels and *Prmt7* knockdown reduced spermatogonial proliferation [5]. In addition, *Prmt7* is reportedly expressed in male gonocytes and spermatogonia [6]. *Prmt7* depletion causes defects in germ cell proliferation as well as a reduced number of primordial germ cells in mice [7]. These findings confirmed the crucial role of *Prmt7* in male germ cell development. Moreover, *Prmt7* is also positively involved in the pluripotency of embryonic stem cells [8]. It mediates the repression of *miR-221-3p* and *miR-221-5p*, which can further target and downregulate the expression of *Oct4*, *Nanog*, and *Sox2*, thus, playing crucial roles in maintaining the pluripotency of mouse embryonic stem cells [9]. Therefore, we propose that *Prmt7* can regulate gene expression through miRNAs, playing roles in spermatogonial proliferation.

It is well known that miRNAs are a class of single-stranded small noncoding RNAs that are approximately 22 nt in length and they post-transcriptionally regulate protein synthesis by degrading mRNA via base pairing to partially complementary sequences in the 3′ UTR of their target mRNAs [10]. Thus, miRNAs play crucial roles in many biological processes, including the cell cycle [11], cell differentiation [12], metabolism [13], cell apoptosis [14], and embryo development [15]. In the gonads, particularly in the testes, miRNAs are ubiquitously present in spermatogonia, Sertoli cells, Leydig cells, and mature spermatozoa. There is growing evidence demonstrating that miRNAs are essential for male germ cell development and differentiation [10,16,17]. For example, *let-7* family miRNAs are highly expressed in type B spermatogonia and primary spermatocytes, highlighting their potential role in spermatogenesis [18]. *MiR-146* levels are decreased with spermatogonial differentiation, and *miR-146* modulates the effects of retinoic acid on spermatogonial differentiation [19]. *MiR-15* downregulation is associated with the abnormal morphology and motility of porcine sperm samples [20]. In addition, *miR-20*, *miR-106a*, *miR-34c*, *miR-221*, *miR-222*, *miR-146a*, and *miR-21* are present and involved in the regulation of mouse spermatogonial stem cells [19,21,22,23,24]. These findings demonstrate the potential roles of miRNAs in male reproduction, especially in germ cell development.

Thus far, the interplay between *Prmt7* and miRNAs is still poorly understood. It has only been reported that *Prmt7* can downregulate *miR-24-2*, *miR-221-3p*, and *miR-221-5p* to maintain the pluripotency of mouse embryonic stem cells [9,25]. However, whether *Prmt7* regulates miRNAs in spermatogonia remains unknown. Thus, in the current study, we aimed to determine if and how *Prmt7* regulates miRNAs in the mouse spermatogonial cell line, GC-1 cells. To this end, we performed *Prmt7* siRNA and negative control siRNA transfection into GC-1 cells, followed by small RNA sequencing. The sequencing results on a subset of DEmiRNAs were verified using qRT-PCR. By comparing the predicted target genes of these DEmiRNAs with our previous transcriptome results, we finally screened seven miRNA/target gene pairs, among which we verified the expression of *Col6a3*, the target gene of *mmu-miR-877-3p*. These results suggested that the proliferation of GC-1 spermatogonia might be mediated through the *Prmt7*/*mmu-miR-877-3p*/*Col6a3* pathway, which helps better understand the role of *Prmt7* in male germ cell development through its miRNA/target gene pathway.

## 2. Materials and Methods

### 2.1. Cell Culture and Treatment

The mouse GC-1 spermatogonial cell line was purchased from iCell (iCell-m022, Shanghai, China). Cells were cultured in a high-glucose Dulbecco’s modified Eagle’s medium (Lonza), supplemented with a 10% fetal bovine serum (Gibco), 100 U/mL penicillin, and 100 μg/mL streptomycin (Invitrogen, Carlsbad, CA, USA) at 37 °C in the humidified incubator with 5% CO_2_. SiRNA transfection was performed using a Lipofectamine RNAiMAX reagent (Invitrogen), according to the manufacturer’s instructions. Briefly, GC-1 cells were seeded into 6-well plates at a density of 1 × 10^5^ cells/well. Then, the cells were incubated in an incubator at 37 °C with 5% CO_2_ overnight. *Prmt7* siRNA or negative control (NC) siRNA at a concentration of 50 nM was transfected into GC-1 cells using Lipofectamine RNAiMAX reagent (Invitrogen). The siRNAs of *Prmt7* (target sequence: TCATGGATGATATGATTAA) and negative control (unpublic) were designed and synthesized by RiboBio (Guangzhou, China). After 48 h of transfection, the cells were collected for further assays.

### 2.2. RNA Extraction and qRT-PCR

Total RNA was extracted from the GC-1 cells 48 h after the siRNA transfection using a TRIzol reagent (Invitrogen). For the mRNA relative expression assay, 1 μg RNA was reverse transcribed using the PrimeScriptTM RT reagent kit (TaKaRa, Dalian, China), according to the manufacturer’s instructions. Then, qRT-PCR was performed with a final volume of the 20 μL reaction system, which contained 10 μL SYBR^®^ Premix Ex TaqTM (TaKaRa), 1 μL cDNA, 0.4 μL forward primer (work concentration 0.2 μM), 0.4 μL reverse primer (work concentration 0.2 μM), 0.4 μL ROX reference dye II, and 7.8 μL sterile distilled H_2_O. β-actin was used as an internal control. For the miRNA relative expression assay, qRT-PCR was performed utilizing the miRNA qRT-PCR SYBR^®^ Kit (Sangon, Shanghai, China) in a 20 μL reaction system, which contained 10 μL master mix, 2 μL cDNA, 0.5 μL forward primer, 0.5 μL reverse primer, 1 μL ROX reference dye (L), and 6 μL RNase-free water. U6 was used as the control. qRT-PCR was carried out on an ABI 7500 Fast Real-Time PCR system (Applied Biosystems, Waltham, MA, USA). The relative expressions of *Prmt7* mRNA and miRNAs were normalized to β-actin and U6 expressions, respectively, and calculated using the 2^−^^ΔΔct^ method [26]. Primer sequences used for qRT-PCR of miRNAs and RNAs are listed in Table 1. Results are presented as fold changes relative to the control.

### 2.3. Protein Extraction and Western Blotting

Proteins were extracted from the GC-1 cells and transfected with siRNA for 48 h. After denaturing, the proteins were electrophoresed on 10% SDS-PAGE gel and then transferred to nitrocellulose membranes. After being blocked with 5% nonfat milk at room temperature for 1 h, the membranes were incubated with primary antibodies at 4 °C overnight. Later, the membranes were washed and further incubated with an HRP-conjugated secondary antibody at room temperature for 1 h. Then, the membranes were developed and visualized on a Tanon-5200 Chemiluminescent Imaging System. The primary antibodies used were PRMT7 (1:1000, #14762) and β-actin (1:1000, #4970) from Cell Signaling Technologies (Danvers, MA, USA). The intensity of each protein band was analyzed using ImageJ software and the protein expression levels were normalized to β-ACTIN.

### 2.4. miRNA Sequencing

Total RNA was extracted from GC-1 cells and transfected with *Prmt7* siRNA or NC siRNA using a TRIzol reagent (Invitrogen). The RNA purity was examined via NanoPhotometer^®^ spectrophotometer (IMPLEN, Westlake Village, CA, USA). The RNA concentration was measured with a Qubit^®^ RNA Assay Kit in a Qubit^®^ 2.0 Flurometer (Life Techonologies, Carlsbad, CA, USA). RNA integrity was tested with a RNA Nano 6000 Assay Kit of Agilent Bioanalyzer 2100 system (Agilent Technologies, Santa Clara, CA, USA).

Small RNA libraries were derived from 3 μg RNA per sample of spermatogonia for each group (*Prmt7* siRNA-transfected group and NC siRNA-transfected group) in triplicate. Sequencing libraries were generated using an NEB-Next^®^ Multiplex Small RNA Library Prep Set for Illumina^®^ (NEB, Ipswich, MA, USA), following the manufacturer’s protocols. The index codes were added to attribute sequences to each sample. Then, the clustering of index-coded samples was performed on a cBot Cluster Generation System using a TruSeq Cluster Kit v3-cBot-HS (Illumina), based on the manufacturer’s instructions. Thereafter, the libraries were sequenced on an Illumina Hiseq 2500 platform. Fifty bp single-end reads were generated (Novogene, Beijing, China). The miRNA sequencing raw data were deposited in NCBI with BioProject accession number PRJNA858498.

### 2.5. miRNA Sequencing Data Analysis

Raw data were firstly processed and further filtered to obtain the clean reads. The small RNA tags were mapped to a reference sequence using Bowtie (bowtie-0.12.9) [27]. Then, their expression and distribution were analyzed. Mapped small RNA tags were used to identify known miRNAs with MiRBase 20.0 as reference. The modified software mirdeep2 (mirdeep2_0_0_5) and srna-tools-cli were used to obtain the potential miRNAs, as well as to draw the secondary structures [28]. In addition, small RNA tags were mapped to the Repeat Masker and Rfam database to remove tags derived from protein-coding genes, repeat sequences, rRNA, tRNA, snRNA, and snoRNA. The novel miRNAs were predicted from the hairpin structure characteristics of a miRNA precursor. The software miREvo (miREvo_v1.1) and mirdeep2 were used integratedly to predict novel miRNAs by exploring the secondary structures, Dicer cleavage sites, and the minimum free energy of small RNA tags unannotated in the former steps [28,29]. The DESeq R package (1.8.3) was used to analyze the differential expression of miRNAs, and adjusted *p* < 0.05 was defined as a significantly differential expression of miRNAs between *Prmt7* siRNA-transfected and NC siRNA-transfected GC-1 cells [30].

### 2.6. Prediction and Functional Enrichment Analyses of miRNA Target Genes

A miRNA target gene prediction was performed using two online tools: miRanda (miRanda-3.3a) and RNAhybrid [31]. The target genes of DEmiRNAs were determined using the intersection predicted with these two databases, followed by being subjected to GO term and KEGG pathway analyses with GOseq and KOBAS, respectively [32,33,34]. GO terms and KEGG pathways were considered significantly enriched when adjusted *p* < 0.05.

### 2.7. Statistical Analysis

The qRT-PCR data were analyzed using GraphPad Prism 5 and the results were presented as mean ± SEM. The statistical significance was calculated through Student’s *t*-test from three independent experiments. *p* < 0.05 was considered statistically significant.

## 3. Results

### 3.1. Validation of Prmt7 Knockdown in GC-1 Cells before miRNA Sequencing

**Figure 1:**
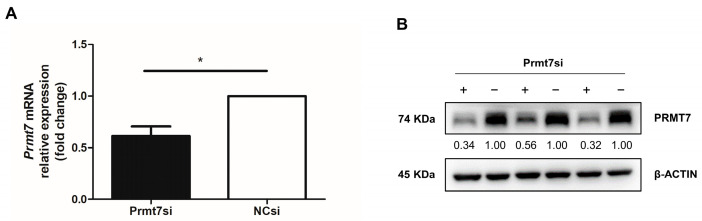
The expression of *Prmt7* in GC-1 spermatogonial cells after *Prmt7* siRNA (Prmt7si) or negative control siRNA (NCsi) transfection for 48 h. (**A**) Relative *Prmt7* mRNA expression detected with qRT-PCR in Prmt7si- and NCsi-transfected GC-1 cells for 48 h. Data from three independent experiments are represented as mean ± SEM. * indicates statistical significance. * *p* < 0.05. (**B**) The protein level of PRMT7 detected with Western blotting in Prmt7si-(shown by “+”) and NCsi-transfected (shown by “−”) GC-1 cells for 48 h. The intensity of each protein band was analyzed using ImageJ software and the pro-tein expression levels were normalized to β-ACTIN.

Our previous work identified that *Prmt7* regulated the proliferation of GC-1 cells (a type of mouse spermatogonial cell line) as the *Prmt7* knockdown reduced their proliferation [5]. However, the underlying molecular mechanisms are still unclear. It is known that miRNAs play essential roles in male germ cell development. Thus, to identify the miRNAs regulated by *Prmt7* and shed light on the mechanisms involved in the proliferation of mouse spermatogonia, we performed small RNA sequencing in GC-1 cells, transfected with *Prmt7* siRNA or negative control (NC) siRNA. Before sequencing, we examined the inhibitory effect of *Prmt7* siRNA in GC-1 cells. The results showed that *Prmt7* expression was significantly reduced after the *Prmt7* siRNA transfection for 48 h at both the mRNA and protein levels (Figure 1A,B).

### 3.2. Analysis of Differentially Expressed miRNAs upon Prmt7 Downregulation

**Figure 2:**
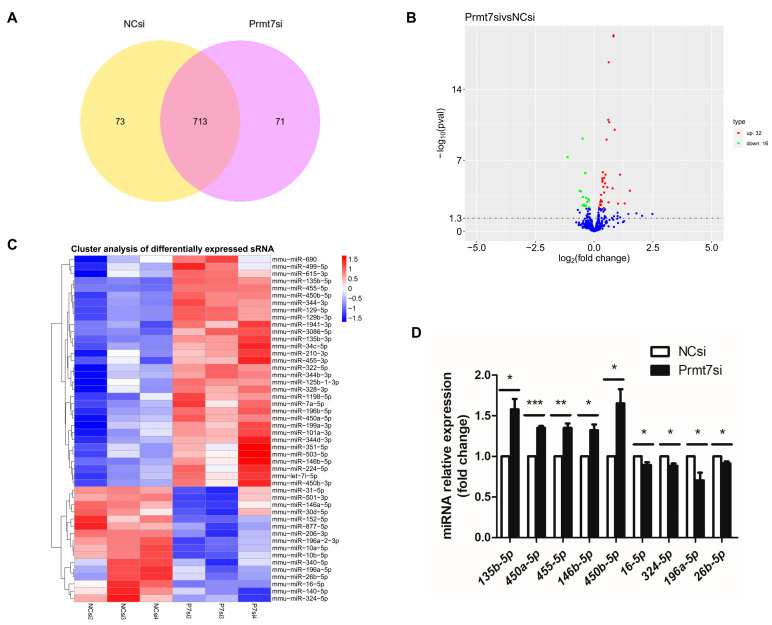
Differentially expressed miRNAs (DEmiRNAs) between *Prmt7* siRNA-transfected and NC siRNA-transfected GC-1 cells. (**A**) Total miRNAs identified in NC siRNA-transfected (left) and *Prmt7* siRNA-transfected GC-1 cells (right). (**B**) Volcano plot of DEmiRNAs in *Prmt7* siRNA-transfected and NC siRNA-transfected GC-1 cells (adjusted *p* < 0.05). Red and green dots represent miRNAs that were upregulated and downregulated, respectively, in the *Prmt7* siRNA group, compared to NC siRNA group. (**C**) Clustered heatmap of DEmiRNAs between the *Prmt7* siRNA-transfected and the NC siRNA-transfected GC-1 cells (adjusted *p* < 0.05). (**D**) Verification of the randomly selected DEmiRNAs using qRT-PCR in the *Prmt7* siRNA group and the NC siRNA group. U6 was used as an internal control. Data from the three independent experiments are represented as mean ± SEM. * indicates statistical significance. * *p* < 0.05, ** *p* < 0.01, and *** *p* < 0.001.

We investigated the miRNAs which were differentially expressed in the *Prmt7* siRNA-transfected GC-1 cells relative to the NC siRNA-transfected GC-1 cells, making a further distinction between upregulation and downregulation. In total, 713 miRNAs were detected in both groups with three replicates (the NC siRNA group and *Prmt7* siRNA group) (Figure 2A). Of the 713 miRNAs, 48 miRNAs were differentially expressed based on an adjusted *p* < 0.05. Of these, 32 and 16 miRNAs were upregulated and downregulated, respectively (Figure 2B). A cluster analysis was also performed to investigate the expression patterns of these differentially expressed miRNAs (DEmiRNAs), as shown in Figure 2C. Prior to further analysis, we verified the DEmiRNAs using an independent method. Specifically, we conducted qRT-PCR on a subset of randomly selected upregulated and downregulated miRNAs. Relative expression levels were normalized to the expression level of U6 snRNA. The qRT-PCR results confirmed the sequencing data and validated that *mmu-miR-135b-5p*, *mmu-miR-450a-5p*, *mmu-miR-455-5p*, *mmu-miR-146b-5p*, and *mmu-miR-450b-5p* were significantly upregulated (*p* < 0.05, *p* < 0.01, and *p* < 0.001), whereas, *mmu-miR-16-5p*, *mmu-miR-324-5p*, *mmu-miR-196a-5p*, and *mmu-miR-26b-5p* were significantly downregulated (*p* < 0.05, Figure 2D) in the *Prmt7* siRNA group, compared to the NC siRNA group. Thus, the miRNA sequencing results were credible.

### 3.3. Functional Enrichment Analysis of Target Genes of DEmiRNAs

**Figure 3:**
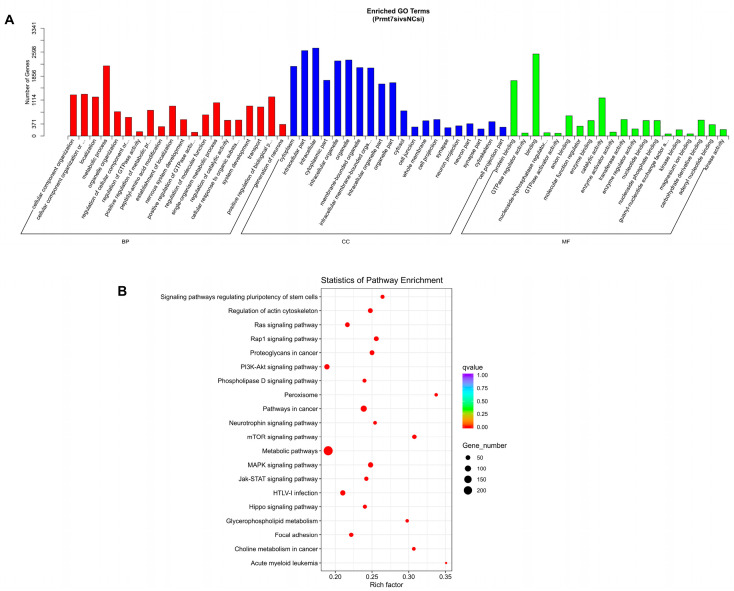
Functional enrichment analysis of target genes. (**A**) GO analysis showed that target genes predicted from DEmiRNAs were involved in 3 classes of GO terms: biological process (BP) shown in red, cellular component (CC) shown in blue, and molecular function (MF) shown in green. The top 20 significantly enriched GO terms are shown. (**B**) KEGG pathway analysis of the target genes predicted from DEmiRNAs. The top 20 enriched KEGG pathways are shown in the bubble diagram. The larger the bubble size, the higher the number of target genes that were enriched. The bubble color changing from red to purple indicates the increasing *p* value.

miRNA target prediction was performed using two online tools: miRanda (miRanda-3.3a) and RNAhybrid. The target genes of DEmiRNAs were determined using the intersection predicted with these two databases. Then, we performed functional enrichment analysis of these intersection target genes. The GO enrichment analysis revealed that these target genes were categorized according to their inclusion in the ontologies biological process (BP), cellular component (CC), and molecular function (MF), resulting in 227 BP terms, 59 CC terms, and 43 MF terms (adjusted *p* < 0.05; Supplementary File S1). The top 20 GO terms for the three ontologies are shown in Figure 3A. The target genes of the DEmiRNAs were mainly enriched in BP terms of the cellular component organization or biogenesis, localization, and metabolic process; in CC terms of cytoplasm and intracellular part; and in MF terms of protein binding, GTPase regulator activity, and binding. Furthermore, the KEGG pathway analysis revealed 165 significantly enriched pathways (adjusted *p* < 0.05; Supplementary File S2) and the top 20 pathways are shown in Figure 3B. The most significantly enriched pathways (top three) associated with *Prmt7* expression were metabolic pathways, pathways in cancer, and the mTOR signaling pathway.

### 3.4. The Possible Molecular Mechanisms Underlying the GC-1 Proliferation Regulated by Prmt7/miRNA/Target Gene

**Figure 4:**
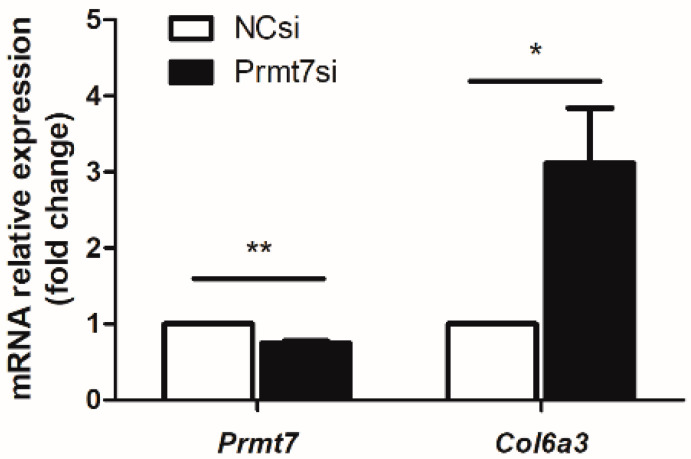
Relative *Prmt7* and *Col6a3* mRNA expression detected using qRT-PCR in *Prmt7* siRNA (Prmt7si)- and negative control siRNA (NCsi)-transfected GC-1 cells for 48 h. Data from three independent experiments are represented as mean ± SEM. * indicates statistical significance. * *p* < 0.05, ** *p* < 0.01.

To identify the possible molecular mechanisms underlying the GC-1 proliferation regulated by the *Prmt7*/miRNA/target gene, we screened the intersection genes between the DEmiRNA target genes and our previously obtained differentially expressed genes (DEGs) from the RNA sequencing of GC-1 cells (NC siRNA group vs. *Prmt7* siRNA group, related raw data were deposited in NCBI with accession PRJNA779471). In total, we obtained 37 target genes as DEGs (Supplementary File S3). Through further screening, seven genes were uniquely regulated by a single miRNA (Table 2). Among these miRNA/target gene pairs, we verified the expression of *Col6a3* (collagen type VI alpha 3), as a target gene of *mmu-miR-877-3p*. The results showed that with the downregulated *Prmt7* expression, the mRNA level of *Col6a3* was upregulated (Figure 4). These data suggest that the proliferation of GC-1 spermatogonial cells might be associated with the *Prmt7*/*mmu-miR-877-3p*/*Col6a3* regulatory pathway.

## 4. Discussion

*Prmt7*, the only type III PRMT, plays a crucial role in the development of mice and humans [35]. Recent studies, including our own, have demonstrated the positively regulatory roles of *Prmt7* in the proliferation of male germ cells [7], which implies its possible function in male reproduction. Growing evidence indicates that male germ cell development relies on miRNAs [16,17]. However, the mechanism by which *Prmt7* regulates the miRNA and target genes in male germ cells remains unclear. In the current study, we examined the miRNA profile in GC-1 cells, a cell line of immortalized mouse spermatogonia, featuring a reduced expression of *Prmt7*, which was achieved by transfection with *Prmt7* siRNA, compared to NC siRNA as a control. As a result, we gained new information regarding the molecular mechanisms of *Prmt7* in spermatogonial proliferation, mediated through miRNA and the target gene.

After the miRNA sequencing of six libraries (two groups, three replicates in each group), we identified 713 miRNAs, including 48 differentially expressed miRNAs (DEmiRNAs), of which 32 were upregulated and 16 were downregulated (adjusted *p* < 0.05). On a subset of these hits, we confirmed the differential miRNA expression using qRT-PCR, in particular, the upregulation of *miR-135b-5p*, *miR-450a-5p*, *miR-455-5p*, *miR-146b-5p*, and *miR-450b-5p,* as well as the downregulation of *miR-16-5p*, *miR-324-5p*, *miR-196a-5p*, and *miR-26b-5p* in *Prmt7* siRNA-transfected GC-1 cells, compared with NC siRNA-transfected GC-1 cells. Among upregulated miRNAs, *miR-146b-5p* inhibits cancers, including gallbladder cancer [36], papillary thyroid cancer [37], nonsmall cell lung cancer [38], colorectal cancer [39], and pancreatic cancer [40]. In addition, we noted the levels of *miR-450a-5p*, which is a proadipogenic miRNA that is able to promote adipogenesis [41]. The roles of these DEmiRNAs are involved in cell proliferation, cancer growth and metastasis, and adipogenesis, which are similar to the functions of *Prmt7*. For example, *Prmt7* deficiency can lead to the defective proliferation of mouse primordial germ cells during the embryonic stage [7]. *Prmt7* downregulation reduced GC-1 mouse spermatogonial proliferation [5]. *Prmt7* overexpression promotes the malignant progression of nonsmall cell lung cancer cells [42] and the growth of renal cell carcinoma [43]. *Prmt7* expression is also associated with the metastasis of breast cancer [44]. Both *Prmt7*-deficient mice and patients with *Prmt7* mutations reportedly exhibit obesity [45,46]. Studies further revealed a repressive role of *Prmt7* in adipogenesis in that *Prmt7* depletion in preadipocytes resulted in enhanced adipogenesis and vice versa [47]. Overall, these DEmiRNAs could be the downstream effectors of *Prmt7*.

Next, we predicted the target genes of these DEmiRNAs and performed a functional enrichment analysis. The GO analysis identified significantly enriched BP terms, including metabolic processes and nervous system development, which were associated with *Prmt7*, since *Prmt7* depletion contributed to reduced oxidative metabolism in skeletal muscle [45]. The loss-of-function of *Prmt7* in mice and humans causes an increased fat mass, suggesting the regulatory role of *Prmt7* in metabolism [35]. Moreover, patients with *Prmt7* mutations exhibit neuron-deficient phenotypes, and, similarly, *Prmt7*-knockout mice exhibit deficient social behaviors [46,48]. In addition, a KEGG pathway analysis for the target genes predicted from these DEmiRNAs was performed. Among the top 20 significantly enriched pathways, we identified pathways in cancer, signaling pathways regulating the pluripotency of stem cells, and the PI3K-Akt signaling pathway, all of which are reportedly associated with *Prmt7* expression. For example, high expression levels of *Prmt7* are correlated with the proliferation and metastasis of breast cancer cells, lung cancer tissues, and clear cell renal cell carcinoma tissues [42,43,44,49]. In clear-cell renal cell carcinoma, *Prmt7* can maintain β-catenin expression to regulate cell proliferation, which demonstrates the possible role of *Prmt7* in regulating the Wnt/β-catenin signaling pathway in cancer. Regarding the involvement of *Prmt7* in the regulation of stem cell pluripotency, *Prmt7* plays a crucial role in maintaining the pluripotency of embryonic stem cells [9,25]. Based on the targeted genes in this pathway, we proposed that *Prmt7* could function in stem cells through the Jak-STAT, MAPK, Wnt, and PI3K-Akt signaling pathways. Moreover, the PI3K-Akt signaling pathway, as a well-known regulator, is involved in many biological processes, such as cell proliferation, apoptosis, metabolism, growth, and survival. This pathway is activated in multiple types of human cancers, including ovarian cancer, brain cancer, breast cancer, and metastatic renal cell carcinoma [50], and maintains the pluripotency of stem cells through the PI3K/Akt/Sox2 axis [51]. Notably, with increased *Prmt7* expression, the phosphorylation of Akt and substrates of mTORC1 were enhanced, suggesting the regulatory roles of *Prmt7* in the Akt/mTORC pathway [52]. Overall, we propose that the miRNAs differentially expressed in response to the expression of *Prmt7* and their target genes in these pathways form a functional signaling pathway in cell proliferation.

To better determine the possible molecular mechanisms underlying the GC-1 proliferation regulated by *Prmt7*/miRNA/target gene, we determined the intersection genes between the DEmiRNA target genes and our previously obtained DEGs from the RNA sequencing of GC-1 cells from the NC siRNA group and *Prmt7* siRNA group. In total, 37 genes in common were obtained, which meant they were considered as both target genes of DEmiRNAs and DEGs. By deleting the nonunique miRNA/target gene pairs, we finally obtained seven genes which were uniquely regulated by single miRNAs. Then, we detected the expression of *Col6a3* (collagen type VI alpha 3, as the target gene of *mmu-miR-877-3p*) in *Prmt7*-downregulated and control GC-1 cells. The results showed *Col6a3* expression was increased with *Prmt7* downregulation, suggesting that *Prmt7* downregulation in mouse spermatogonia might function through miR-877-3p/*Col6a3*. It has only been reported that *Col6a3* is associated with cancer [53,54], muscular dystrophy [55,56], and obesity [57,58], which exhibits the possible similar roles of *Prmt7*. However, thus far, there is no evidence showing the role of *mmu-miR-877-3p* or *Col6a3* in male reproduction. Therefore, further study should be performed in the future to help better understand the regulatory roles of *Prmt7* in male reproduction.

## 5. Conclusions

In summary, to identify the miRNAs regulated by *Prmt7* in spermatogonia and further explore the possible pathway involved in *Prmt7* regulation in mouse spermatogonial cell line GC-1 cells, we performed small RNA sequencing, as well as integrated analyses with previous RNA sequencing data for *Prmt7* siRNA and NC siRNA-transfected GC-1 cells. As a result, we identified 48 differentially expressed miRNAs, among which 32 and 16 miRNAs were upregulated and downregulated, respectively. The intersection between the target genes of these DEmiRNAs and previously obtained DEGs using RNA sequencing was further screened and seven miRNA/target gene pairs were obtained. Further detection suggested that *Prmt7* downregulation in mouse spermatogonia might function through *miR-877-3p*/*Col6a3*. These data will provide insight into the further exploration of the roles of *Prmt7* in male germ cell development and in male reproduction.

## Figures and Tables

**Table 1:** Primers used for qRT-PCR assessing the relative expression of RNAs and miRNAs in mouse GC-1 spermatogonia.

miRNA		Primer Sequences
*Prmt7*	Forward primer	GGAGATTGCCAGGTCATCCT
	Reverse primer	GAAGTCAGCCCCTGCAGTAA
*Col6a3*	Forward primer	GAGATGGGGTTGGCAGTGAA
	Reverse primer	GCATTGATCAACGGAGTCGC
*β-actin*	Forward primer	TGTGCTGTCCCTGTATGCCTCT
	Reverse primer	TAGATGGGCACAGTGTGGGTGA
*mmu-miR-135b-5p*	Forward primer	CGCGTATGGCTTTTCATTCCTATGTGA
*mmu-miR-450a-5p*	Forward primer	CGCGCTTTTGCGATGTGTTCCTAATAT
*mmu-miR-455-5p*	Forward primer	CGCTATGTGCCTTTGGACTACATCG
*mmu-miR-146b-5p*	Forward primer	CCGCTGAGAACTGAATTCCATAGGCT
*mmu-miR-450b-5p*	Forward primer	GCGCGTTTTGCAGTATGTTCCTGAATA
*mmu-miR-16-5p*	Forward primer	CCGTAGCAGCACGTAAATATTGGCG
*mmu-miR-324-5p*	Forward primer	TATATACGCATCCCCTAGGGCATTGG
*mmu-miR-196a-5p*	Forward primer	CGCGTAGGTAGTTTCATGTTGTTGGG
*mmu-miR-26b-5p*	Forward primer	GCGCGTTCAAGTAATTCAGGATAGGT

β-actin and U6 were served as controls of RNAs and miRNAs, respectively.

**Table 2:** Intersection genes by combining target genes of differentially expressed miRNAs and differentially expressed genes between NC siRNA group and *Prmt7* siRNA group GC-1 cells.

Intersection Gene	Log2 FC	miRNA	Log2 FC
*Fut7*	−1.62	*mmu-miR-135b-3p*	1.29
*Atp2c2*	1.25	*mmu-miR-140-5p*	−0.22
*Gm14406*	1.38	*mmu-miR-30d-5p*	−0.40
*Cracdl*	1.03	*mmu-miR-31-5p*	−0.57
*Hmcn2*	1.12	*mmu-miR-324-5p*	−0.50
*Lefty2*	1.69	*mmu-miR-501-3p*	−0.49
*Col6a3*	1.08	*mmu-miR-877-5p*	−0.49

## Data Availability

The data presented in this study are available on request from the corresponding author.

## Supplementary Materials

The following supporting information can be downloaded at: https://www.mdpi.com/article/10.3390/life12081194/s1, Supplementary File S1: GO terms of target genes; Supplementary File S2: KEGG pathways of target genes; Supplementary File S3: intersection gene screening.
